# Targeting hexokinase 2 to enhance anticancer efficacy of trichosanthin in HeLa and SCC25 cell models

**DOI:** 10.5599/admet.2455

**Published:** 2024-09-22

**Authors:** Yan Zhou, Maoxin Ran, Wenying Shan, Kaifang Wang, Ou Sha, Kin Yip Tam

**Affiliations:** 1 Faculty of Health Sciences, University of Macau, Taipa, Macau; 2 School of Dentistry, Shenzhen University Medical School, Shenzhen, China

**Keywords:** Ribosome-inactivating protein, glycolysis, drug combination, synergistic effect, cancer therapy

## Abstract

**Background and purpose:**

Trichosanthin (TCS) is a plant-based ribosome-inactivating protein exhibiting a range of pharmacological properties, including abortifacient and anticancer. However, the routine clinical use in cancer treatment was hampered by its antigenicity. Hexokinase 2 (HK2) is a pivotal regulator of glycolysis, where aberrant expression is observed in many cancers. This study investigates the anticancer effects and mechanisms of TCS in combination with benserazide (Benz), a HK2 inhibitor, in Hela and SCC25 cancer models.

**Experimental approach:**

MTT, colony-formation and cell cycle assays were performed to assess the cytotoxic effects of TCS and Benz in HeLa and SCC25 cells. Seahorse assay, western blotting, flow cytometry analysis and RNA sequencing were employed to investigate the pharmacological effects of the combo treatment. SCC25 cell xenograft mouse model was established for *in vivo* efficacy study.

**Key results:**

Combined use of TCS and Benz exhibited synergistic anticancer effects in both Hela and SCC25 cell models. The observed synergistic effects were attributed to the modulation of glycolysis by targeting HK2, leading to reduced lactate production and increased ROS accumulation which further inhibited colony formation and cell cycle progression, as well as triggered apoptosis. Moreover, this combination effectively inhibited NFκB/ERK signalling pathways, which were found to be significantly activated upon single use of TCS. It was found that the combination significantly suppressed the tumour growth in SCC25 cell xenograft mouse model.

**Conclusion:**

Our findings suggested that targeting HK2 and modulating glycolysis may offer a promising avenue for improving the therapeutic outcomes of TCS-based anticancer treatments.

## Introduction

Cancer, characterized by uncontrolled cell growth and proliferation, is one of the leading causes of death worldwide [1]. Aberrant cellular metabolism has been recognized as a hallmark of cancer, and one prominent metabolic alteration is the upregulation of glycolysis, a phenomenon known as the Warburg effect, which involves increased glycolysis and lactate production even in the presence of sufficient oxygen [2,3]. This metabolic reprogramming enables cancer cells to sustain their energy demands and support rapid proliferation [4,5]. Hexokinase 2 (HK2), a key enzyme in the glycolytic pathway, plays a critical role in facilitating glucose metabolism by phosphorylating glucose to glucose-6-phosphate, initiating its entry into glycolysis pathway [6]. Aberrant upregulation of HK2 has been observed in various cancer types, promoting tumour growth, progression, metastasis, and drug resistance [7]. Inhibiting HK2 activities could directly target the metabolic alterations in cancer cells, particularly disrupting the aberrantly facilitated glycolysis. This disruption can lead to a reduced availability of glycolytic intermediates and energy production that benefiting cancer cell proliferation [8]. Consequently, targeting HK2 has emerged as a promising adjunctive therapy to disrupt the metabolic rewiring of cancer cells [9,10].

Recently, there has been a surge of interest in natural compounds as potential anticancer agents [11,12]. One such bioactive compound is trichosanthin (TCS), which derived from the root tubers of Chinese medicinal herb *Trichosanthes kirilowii* [13]. TCS possesses molecular weight of 27 kDa and is composed of a single polypeptide chain containing 247 amino acid residues [14]. As a type 1 ribosome-inactivating protein (RIP), TCS can selectively cleave the conserved sarcin/ricin loop (SRL) in the large ribosomal RNA (rRNA) subunit, which leads to the inhibition of protein synthesis, ultimately resulting in cell apoptosis [15]. TCS has gained prominence for its *in vitro* and *in vivo* anticancer properties by demonstrating multi-faceted mechanisms including the inhibition of protein synthesis, suppression of tumour angiogenesis, triggering apoptosis, affecting cell cycle, and modulating immune responses in numerous types of cancer cells [15-19]. The multitude of anticancer properties makes it a potential therapeutic candidate for further research and evaluation.

Despite the promising potential, clinical adoption of TCS as a monotherapy to anticancer has been limited, attributing to its non-specificity and the potential toxicity at high doses [15,20]. Therefore, it would be of great interest to develop novel strategies for enhancing the anticancer effects of TCS while lowering the efficacious dose to minimize toxic effects, which may facilitate successful clinical translation. To date, several studies have indicated that TCS could be utilized as a constituent of a drug cocktail or combination therapy to boost its efficacy [21-23]. It has been reported that TCS synergized imatinib to suppress the NF-κB signaling pathway resulting in enhanced inhibitory effects in chronic granulocytic lineage leukaemia cells [24]. As far as we are aware, little, if any, work had been undertaken to explore the anticancer effects in the combined use of TCS and HK2 inhibitor.

In the present study, we explore the innovative and rational combination of TCS with benserazide hydrochloride (Benz), a HK2 inhibitor, using HeLa (human cervical cancer) and SCC25 (human oral squamous cell carcinoma) cells as cancer models. As a Food & Drug Administration (FDA) approved compound used along with levodopa to treat Parkinson’s disease, Benz was previously reported to block HK2 enzymatic activity with tolerable toxicity [25]. By combining TCS with Benz, we seek to evaluate the anticancer synergism, assess the metabolic alterations, and investigate the molecular mechanisms in response to the designed combination regimen.

## Experimental

### Cell lines and cell culture

HeLa and SCC25 cell lines were purchased from the American Type Culture Collection. Human fibroblast cell line was a gift from Prof. Guokai Chen (Faculty of Health Sciences, University of Macau). All cells were grown in full media containing DMEM (Gibco, Thermo Fisher Scientific, USA) with 10% fetal bovine serum (FBS), maintained in a 37 °C, 5% CO_2_ atmosphere incubator.

### Materials

TCS was prepared by molecular cloning as previously described [18]. Benz was purchased from Aladdin (Shanghai, China). Primary antibodies against Hexokinase II (C64G5) (Cat#2867), NF-κB p65 (D14E12) (Cat#8 242), Phospho-NF-κB p65 (Ser536) (93H1) (Cat#3033), p44/42 MAPK (Erk1/2) (Cat#9102), phospho-p44/42 MAPK (Erk1/2) (Thr202/Tyr204) (Cat#9101), caspase-3 (Cat#9662), cleaved Caspase-3 (Asp175) (Cat#9661), and β-actin (Cat#4967S) were purchased from Cell Signaling Technology (CST, USA). PCNA Antibody (PC10) (Cat#sc-56) was purchased from Santa Cruz Biotechnology (SCBT, Santa Cruz, CA, USA). The secondary antibodies anti-mouse IgG, HRP-linked antibody (Cat#7076) and anti-rabbit IgG, HRP-linked antibody (Cat#7074) were purchased from Cell Signalling Technology (CST, USA).

### Cell viability MTT assay

Cells were seeded onto 96-well plates and incubated overnight to allow cell attachment, then culture medium were refreshed with medium containing desired drug and treated for additional 72 h. Subsequently, the medium was discarded and refreshed with 100 μl culture media containing 0.5 mg/ml MTT (3-(4,5-dimethyl-2-thiazolyl)-2,5-diphenyl-2-H-tetrazoliumbromide). After 4 h incubation, the supernatant was removed and 100 μl DMSO were added to fully dissolve the formazan crystals. At last, the O.D. values of each well were read at 570 nm by the Spectra Max M5 Microplate Reader (Molecular Devices, USA).

### Chou-Talalay drug synergy assessment

Drug synergy was assessed by combination index (CI), equation (1):


(1)

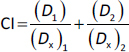



where *D*_1_ and *D*_2_ were the drug combination doses to achieve a specific effect. While the individual drug dose which achieves the equal effect were represented as (*D*_x_)_1_ and (*D*_x_)_2_. The TCS and Benz were combined at a non-fixed ratio. CI < 0.9 indicates synergism, 0.9 < CI < 1.1 indicates additivity, and CI > 1.1 indicates antagonism [26-28]. The CalcuSyn software (Biosoft, Ferguson, MO, USA) was used to calculate CI values and the fraction of affected (*F*_a_), which was defined by equation (2):


(2)

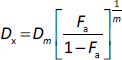



*D_m_*, represents the concentration (*e.g. IC*_50_) at which 50 % inhibition of cancer cells occurs. The slope coefficient (*m*) determines the shape of the dose-effect curve in the dose-effect relationship. Value of m equal 1 indicates a hyperbolic dose-effect curve, while values greater than 1 and less than 1 represent sigmoidal and flat sigmoidal curves, respectively. The CI values were plotted against the fraction affected (*F*_a_), and the data were also evaluated using the Combination Index-Isobologram equation. Only CI values obtained at *F*_a_ values > 0.5 can be considered as relevant [28].

### Seahorse XFe96 analysis

The glycolytic function parameter extracellular acidification rate (ECAR) was measured using a Seahorse XFe96 Analyzer (Agilent Technologies, USA). Approximately 25,000 cells per well were seeded in XF96-well plates, cells were treated with TCS and Benz alone or in combination for 18 h. The Glycolysis Stress Test was utilized to analyse glycolysis activity. Briefly, the culture medium was replaced with XF assay medium containing 2 mM glutamine and no glucose, and the cells were incubated at 37 °C in a CO_2_-free incubator for 1 h. The Glycolysis Stress Test kit (Agilent Technologies, USA) involved sequentially adding glucose (10 mM), oligomycin (1 μM), and 2-deoxyglucose (2-DG, 50 mM). After each injection, the ECAR was measured, and the data were normalized to the cell number in each well. Data analysis was performed in Wave software (Agilent Technologies, USA).

### Measurement of lactate secretion

To measure the extracellular lactate secretion, cells were seeded in 6-well plates and treated with either TCS or Benz alone, or in combination for 24 h. The supernatants of each sample were collected and then centrifuged at 8000 rpm for 5 min to remove any cellular debris. The lactate concentration in the supernatant was measured using the Nova BioProfile FLEX analyser (Nova Biomedical, USA), which utilized a combination of enzymatic and optical methods to detect and quantify lactate levels.

### Colony formation assay

Cells were seeded into 6-well plates at a respective optimized density from 100 to 300 cells per well. After 24 h incubation, the culture medium was refreshed with medium containing designed individual or combination of compounds treatment. After 72 h treatment, the medium was replaced with compound-free culture medium every 3 to 4 days. When the control wells which with no treatment were filled with colonies which would take about 2 weeks, all wells were added with 95 % ethanol for 15 min to fix the colonies, and then stained with 0.1 % crystal violet (Sigma Aldrich, USA). The image J software package was used to count colonies in each well.

### Cell cycle analysis

Cells were seeded in 6-well plates, after 24 h attachment, cells were exposed to drug treatment for 24 h. Then cells were harvested, washed and fixed with 70 % ethanol for at 4 °C refrigerator for 24 h. Then cell samples were washed with 1X PBS and incubated with 50 l of 100 g/ml RNAase for 30 min, followed by adding 200 l cold propidium iodide (Sigma Aldrich, USA) (50 g/ml) and incubated on ice for 30 min. Finally, the cell cycle distribution was analysed using a Cyto-Flex flow cytometer (Beckman Coulter, Indianapolis, IN, USA).

### Measurement of reactive oxygen species

Intracellular levels of reactive oxygen species (ROS) were assessed using the Reactive Oxygen Species Assay Kit (Beyotime, China). The assay utilized 2',7'-dichlorofluorescein diacetate (DCFH-DA) as a fluorescent probe. Briefly, cells were seeded at a density of 3×10^4^ cells per well in 96-well plates and incubated overnight at 37 °C. Subsequently, the cells were treated with 10 μM DCFH-DA for 30 min, followed by three washes with culture medium without FBS. After drug treatment, the fluorescence intensity was measured using the SpectraMax M5 microplate reader with excitation wavelength set at 488 nm and emission wavelength set at 525 nm. The results were expressed as the fold change in fluorescence intensity relative to the control group.

### JC1 assay to detect mitochondrial depolarization

Flow cytometry assay was performed to detect the mitochondrial membrane potential (MMP, Δ*ψm*) using JC-1 staining. Cells were washed twice with PBS after designed treatment, then incubated with whole culture medium containing appropriate concentration of 5,5’,6,6’-tetrachloro-1,1’,3,3’-tetraethylbenzimidazolylcarbocyanine iodide (JC-1) (Sigma Aldrich, USA) for 15 min at a 37 °C incubator. Then the red and green fluorescence signals of cells were analysed by the Cyto-Flex flow cytometer (Beckman Coulter, Indianapolis, IN, USA).

### Early and late apoptosis detection assay

Cell apoptosis proportion was analysed by Annexin V-FITC/ Propidium Iodide (PI) (Invitrogen, CA, USA) dual staining. Cell samples were harvested using TrypLE (Gibco, Thermo Fisher Scientific, USA) and washed with cold PBS for two times. Then cell pellets were immediately suspended in 500 μL Binding Buffer which containing 5μL of Annexin V-FITC and 10 μL of PI (FITC Annexin-V Apoptotic Detection Kit, Bio legend). After 15 min incubation at room temperature, cell samples were analyzed by the Cyto-Flex flow cytometer (Beckman Coulter, Indianapolis, IN, USA).

### Western blot

Target proteins were separated on 10 % SDS-PAGE, then electrophoretically transferred to a nitrocellulose membrane at 4 °C for 12 h by the Trans-Blot System (Bio-Rad, USA). The membrane was blocked in 5 % BSA for 1 h and then washed 3 times with TBST before probed with specific primary antibodies overnight at 4 °C. After that, the membrane was washed and reacted with appropriate secondary antibodies which were used to detect the target proteins. Finally, the proteins of samples were visualized by a Chemidocs MP Imaging System (Bio-Rad, USA).

### RNA-Sequencing analysis

The total RNA of HeLa cells after designed treatments were extracted following the manufacturing instructions. Briefly, after washing with PBS, cells in 6-well plates were added with 500 μL/well RNAiso Plus reagent and vigorously pipetted to dissolve completely. Then samples were sent to the Novogene (Beijing, China) for performing RNA sequence analysis.

### Cellular ATP Measurement

Cells were prepared according to the manufacturer's instructions provided by the cellular ATP test kit (Beyotime, China). Briefly, triplicates of samples or ATP standard solutions were dispensed into a 96-well microplate. The ATP assay reagent from the kit was added to each well and mixed thoroughly. Luminescence signal was measured using a luminometer-equipped SpectraMax M5 Microplate Reader. ATP levels were recorded and normalized to sample protein concentration.

### Hexokinase activity assay

The hexokinase activities were measured using a Hexokinase (HK) Activity Assay Kit (Solarbio, China). Experiments were conducted as manufacturer instructions. Finally, the absorption values of the cell samples were read at 340 nm by the Spectra Max M5 Microplate Reader (Molecular Devices, USA).

### Isolation of nuclei from cells

The separation of nuclei from cells was conducted as previously published protocol [29]. In brief, the dissociated cells were transferred to a 1.5 mL microcentrifuge tube kept on ice. After centrifugation, the supernatant was removed, and the cell pellet was resuspended in ice-cold hypertonic buffer containing 0.1 % NP-40. The sample was gently mixed and triturated, followed by centrifugation at 10,000 rpm for 10 min. The supernatant was discarded, leaving behind white pelleted nuclei, which were than isolated from the yellowish pellets of whole cells. The isolated nuclei were further utilized for a variety of downstream western blotting experiments.

### In vivo xenograft study

The Animal Research Ethics Committee of the University of Macau authorized and guided all animal studies, which followed the approved protocol (UMARE-008-2022; approved date: 17-05-2022) that was in compliance with the U.K. Animals (Scientific Procedures) Act, 1986 and associated guidelines. Female nude mice, aged 6-8 weeks, were procured from the Animal Core of the University of Macau and kept in a pathogen-free environment. To establish xenograft tumour s, SCC25 cells (around 8×10^6^ cells in 100 μl PBS) were subcutaneously injected into the right flank of each mouse. Once the tumour s reached a volume of approximately 100 mm^3^, the mice were randomly divided into five groups (*n* = 6 per group) and treated with vehicle (PBS containing 10 % PEG), TCS (1 mg/kg), Benz (500 mg/kg), and TCS combined with Benz by intraperitoneal injection every other day for 19 days. The body weight and general conditions of mice were monitored every two days. tumour s was measured every two days with a calliper and the tumour volumes were calculated by applying the formula which depicted as *V* = (*W*^2^*L*)/2, where *V* is tumour volume, *W* is tumour width and *L* is tumour length.

### Bioinformatics analysis

Normalized HK2 protein expression values were downloaded from the Clinical Proteomic tumour Analysis Consortium (CPTAC) data portal. Pan-cancer molecular subtypes were revealed by mass-spectrometry-based proteomic characterization of more than 500 aggressive human cancers. The mRNA expressions of HK2 were obtained from the R package TCGAbiolinks. TPM (transcripts per kilobase per million) was used to quantify the mRNA expression and to compare the gene expression level between tumour s and their paired normal tissues.

### Statistical analysis

All experiments were independently repeated at least three times, except for individual tumour measurements. For the statistical difference comparisons between two groups, two-tailed Student’s t-test was used. For comparisons among multiple groups, data were analysed using ANOVA. Statistical data were shown as mean ± SE (standard error) (*n* = 3 independent experiments). Data were analysed by GraphPad Prism8 Software and the p value less than 0.05 was considered significant (ns indicates non-significant, * represents *P*< 0.05, ** represents *P*<0.01, *** represents *P*<0.001, **** represents *P*< 0.0001).

## Results

### HK2 expressions were significantly enhanced in malignant tumour s

A number of tumour s with high expression of HK2 have been associated with malignant properties [30-33]. To gain insights into the potential roles of HK2 in tumour malignancy, particularly in cervical cancer and oral squamous cell carcinoma (OSCC), databases from the Cancer Genome Atlas (TCGA) and the National Cancer Institute’s Clinical Proteomic Tumour Analysis Consortium (CPTAC) were used to analyse the transcriptional expression and protein level of HK2.

**Figure 1.**
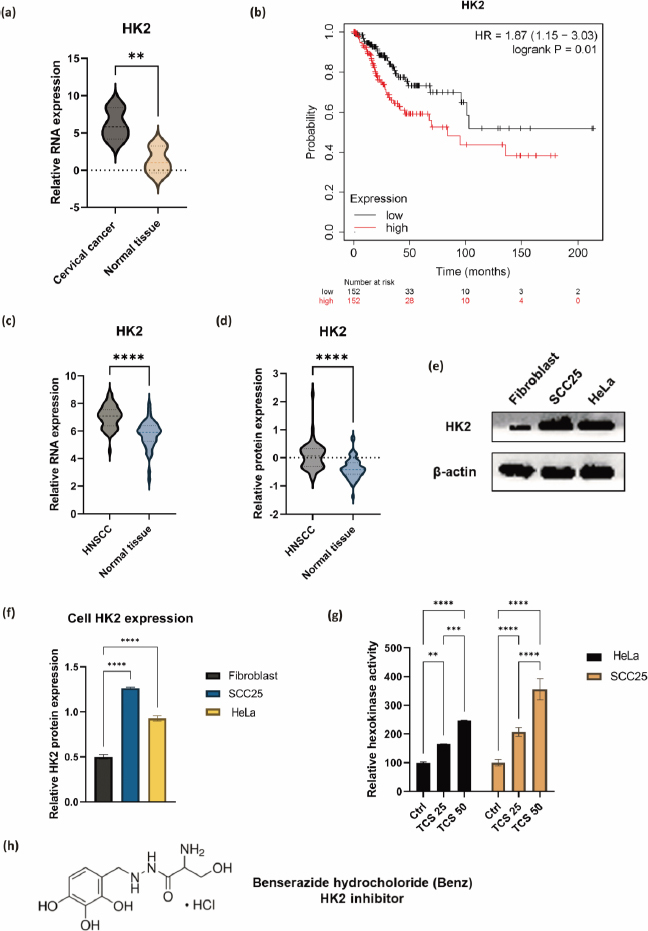
Investigation of HK2 expression and its potential clinical significance in malignant tumour s. **(a)** The relative mRNA expressions of HK2 in cervical carcinoma tissues and adjacent normal tissues. **(b)** Kaplan–Meier survival curves of patients with cervical cancer stratified by high and low HK2 transcription expression levels (*P* < 0.05). **(c, d)** The relative mRNA and protein expression levels of HK2 in HNSCC tissues and adjacent normal tissues. **(e)** Western blot detection of HK2 protein expression on three cell lines, including normal fibroblast, SCC25, and HeLa. **(f)** Quantified data were presented as mean ± SE (*n* = 3). **(g)** The hexokinase activities in HeLa and SCC25 cells under 25 and 50 μg/mL TCS treatments. **(h)** Chemical structure of HK2 inhibitor, benserazide hydrochloride. **represents *P*<0.01, **** represents *P* < 0.0001.

Compared with adjacent normal tissues, expressions of HK2 mRNA were significantly higher in cervical cancers (Figure 1a). Subsequent Kaplan-Meier survival analysis showed that HK2 expression was negatively correlated with the overall survival probability of patient with cervical carcinoma (Figure 1b). Unfortunately, there is a scarcity of data specifically on oral squamous cell carcinoma (OSCC). Nevertheless, we investigated head and neck squamous cell carcinoma (HNSCC), which is a broader term that covers squamous cell carcinomas from different parts of the head and neck area, including the oral cavity. Both mRNA and protein expression levels were pronounced higher in HNSCCs than normal tissues (Figures 1c and 1d).

Moreover, the enhanced HK2 protein expressions on HeLa and SCC25 cell lines compared to normal human fibroblast cells were validated *via* western blotting (Figures 1e and 1f). In addition, 6 h TCS treatments significantly facilitated hexokinase activities in both HeLa and SCC25 cells, indicating TCS single treatment enhanced HK2 activities (Figure 1g). With these in mind, the HK2 inhibitor Benz (Figure 1h) was used in combination with TCS to evaluate the anticancer effects in HeLa and SCC25 cell models.

### Combined use of TCS and Benz demonstrated strong anticancer synergism in HeLa and SCC25 cells

**Figure 2.**
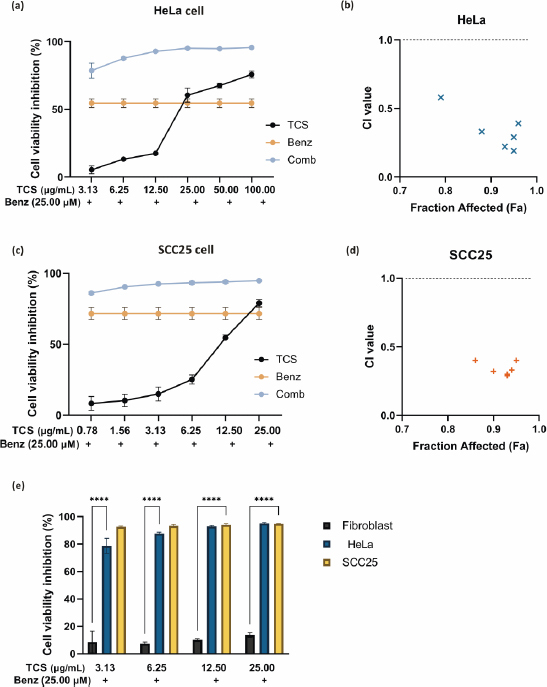
Drug synergy of TCS and Benz combination in HeLa and SCC25 cells. **(a)** HeLa cell viability results after 72 h treatments of TCS or Benz alone and in combination. **(b)** Plots of CI values for combined treatments of TCS and Benz at different concentrations in HeLa cells. CI values < 0.9, 0.9 < CI < 1.1 or > 1.1 indicates drug synergism, additive, or antagonism, respectively. **(c)** Cell viability inhibition in SCC25 cells under 72 h treatments. **(d)** *F*_a_-CI plots in SCC25 cells. **(e)** Evaluating the cell growth inhibition of combined TCS and Benz treatment in HeLa and SCC25 cancer cells compared to normal fibroblasts. Data were expressed as mean ± SE (*n* = 3); ** represents *P*<0.01, **** represents *P*< 0.0001.

The *IC*_50_ values of TCS and Benz in HeLa and SCC25 cells under 72 h treatments were determined by MTT assays (Table 1). To examine the drug synergy, HeLa cells were treated with different concentrations of TCS and Benz, either alone or in combination, for 72 h, followed by MTT assay. Compared with each single compound treatment, the combo significantly inhibited cell survival rate (Figure 2a). Combination index (CI) described by Chou & Talalay [27] was then employed to analyse drug synergism. Each combined treatment of TCS and Benz demonstrated a strong synergistic effect with potent anticancer efficacy in HeLa cells, indicated by CI values less than 1 while fraction affected (*F*_a_) values achieved more than 0.5 as shown in Figure 2b and Table 2. Consistently, similar results were obtained in SCC25 cell model (Figures 2c and 2d, and Table 3). Besides, each combination exhibited pronounced cell viability inhibition in HeLa and SCC25 cells with only relatively mild effects in human normal fibroblasts (Figure 2e).

**Table 1.** The *IC*_50_ values of TCS and Benz in HeLa and SCC25 cells.^a^

Cell line	TCS *IC*_50_ / μg mL^-1^	Benz *IC*_50_ / μM
HeLa	24.08 ± 2.29	29.34 ± 0.12
SCC25	12.28 ± 1.90	18.31 ± 1.50

^a^Data was presented as mean ± SD (*n* = 3).

**Table 2.** Treatment concentrations, *F*_a_ values and CI values for TCS and Benz combination in HeLa cells (*n* = 3).

Treatment group	TCS *IC*_50_ / μg mL^-1^	Benz *IC*_50_ / μM	*F*_a_	CI
1	3.13	25.00	0.79	0.58
2	6.25	25.00	0.88	0.33
3	12.50	25.00	0.93	0.22
4	25.00	25.00	0.95	0.19
5	50.00	25.00	0.95	0.29
6	100.00	25.00	0.96	0.39

**Table 3.** Treatment concentrations, *F*_a_ values and *CI* values for TCS and Benz combination in SCC25 cells (*n* = 3).

Treatment group	TCS *IC*_50_ / μg mL^-1^	Benz *IC*_50_ / μM	*F*_a_	CI
1	0.78	25.00	0.86	0.40
2	1.56	25.00	0.90	0.32
3	3.13	25.00	0.93	0.29
4	6.25	25.00	0.93	0.30
5	12.50	25.00	0.94	0.33
6	50.00	25.00	0.95	0.40

### Combination of TCS and Benz significantly decreased cellular glycolysis and ATP levels

**Figure 3.**
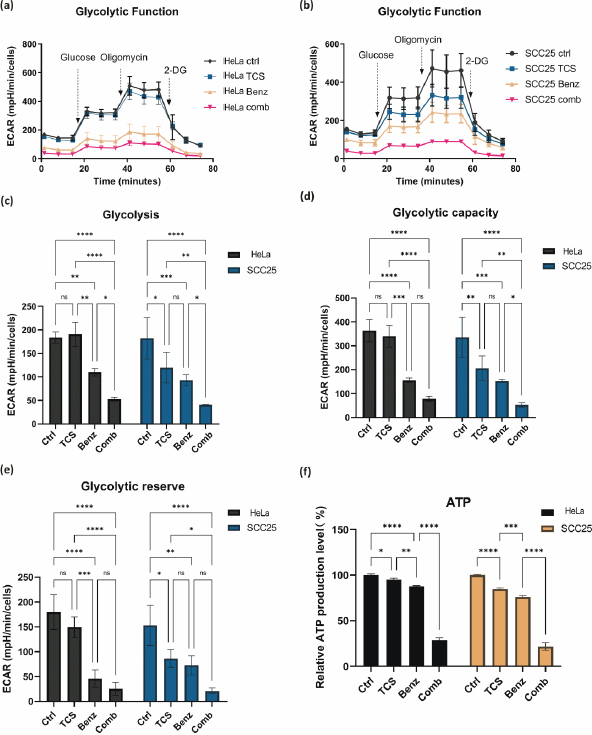
Changes in metabolic and energy parameters in HeLa and SCC25 cells exposed to TCS and Benz combined treatments in HeLa and SCC25 cells. **(a, b)** ECAR profiles of drug-treated HeLa and SCC25 cells under three sequential injections of glucose (10 mM), oligomycin (1 μM), and 2-deoxyglucose (2-DG, 50 mM). ECAR values were normalized to cell numbers. **(c, d, e)** ECAR data were quantified to indicate basal glycolysis, glycolysis capacity, and glycolysis reserve in HeLa and SCC25 cells. **(f)** Measurement of cellular ATP amount in HeLa and SCC25 cells. Data were presented as mean ± SE (*n* = 3); ns indicates non-significant, * represents *P*< 0.05, ** represents *P*<0.01, ***represents *P*<0.001, **** represents *P*< 0.0001.

Next, the changes of metabolic profile associated with glycolysis were measured by Seahorse assay. HeLa cells were treated with 50 μg/mL TCS and 50 μM Benz, alone or in combined, for 18 h prior to measuring extracellular acidification rate (ECAR). SCC25 cells were treated with 25 μg/mL TCS and 25 μM Benz, alone or in combined, for 18 h, followed by Seahorse assay. Compared to single treatment of TCS or Benz, the combined treatment decreased ECAR profiles in both HeLa and SCC25 cells, suggesting attenuated glycolytic function (Figure 3a, b). Quantitative analyses on the ECAR profile data showed that this combination markedly reduced basal glycolysis, glycollysis capacity, and glycolysis reserve in HeLa and SCC25 cells (Figure 3c, 3d and 3e). Moreover, 24 h of TCS combined with Benz treatments resulted in dramatic decreasing of cellular ATP production in both cell lines (Figure 3f).

### TCS and Benz combination lowered lactate secretion and suppressed colony-forming ability

**Figure 4.**
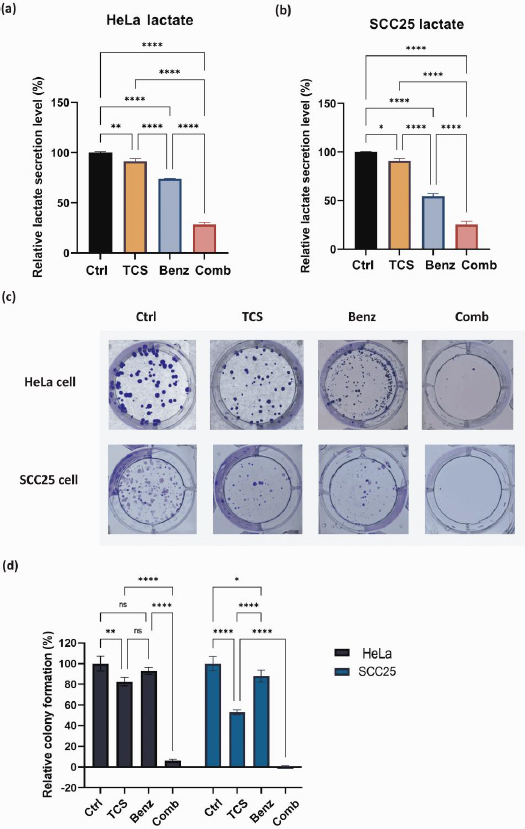
Lactate secretion and colony formation in HeLa and SCC25 cell models. **(a, b)** Quantification of extracellular lactate level after 24 h treatment. HeLa cells were treated with 50 μg/mL TCS and 50 μM Benz, alone or in combined. SCC25 cells were treated with 25 μg/mL TCS and 25 μM Benz, alone or in combined. **(c, d)** Colony formation and quantification results. HeLa cells were treated with 12.50 μg/mL TCS and 5 μM Benz, alone or in combined. SCC25 cells were treated with 6.25 μg/mL TCS and 2.5 μM Benz, alone or in combined. After 72 h drug exposure, cells were maintained in drug-free culture medium to form colonies. Data were presented as mean ± SE (*n* = 3); ns indicates non-significant, * represents *P*< 0.05, ** represents *P*<0.01, ***represents *P*<0.001, **** represents *P*< 0.0001.

The byproduct of glycolysis, lactate, was measured and quantified by Nova BioProfile FLEX analyzer. After 24 h, the TCS-Benz combo significantly reduced the extracellular lactate levels in both HeLa and SCC25 cells (Figures 4a and 4b). Lactate generated *via* glycolysis was known for promoting cancer cell colony formation and survival by providing energy and creating an acidic microenvironment. In HeLa and SCC25 cells, TCS-Benz combo significantly reduced colony size and numbers as compared with single treatment, indicating the proliferative capacity of cancer cells was effectively attenuated (Figures 4c and 4d).

### TCS combined with Benz induced cell cycle arrest in HeLa and SCC25 cells

**Figure 5.**
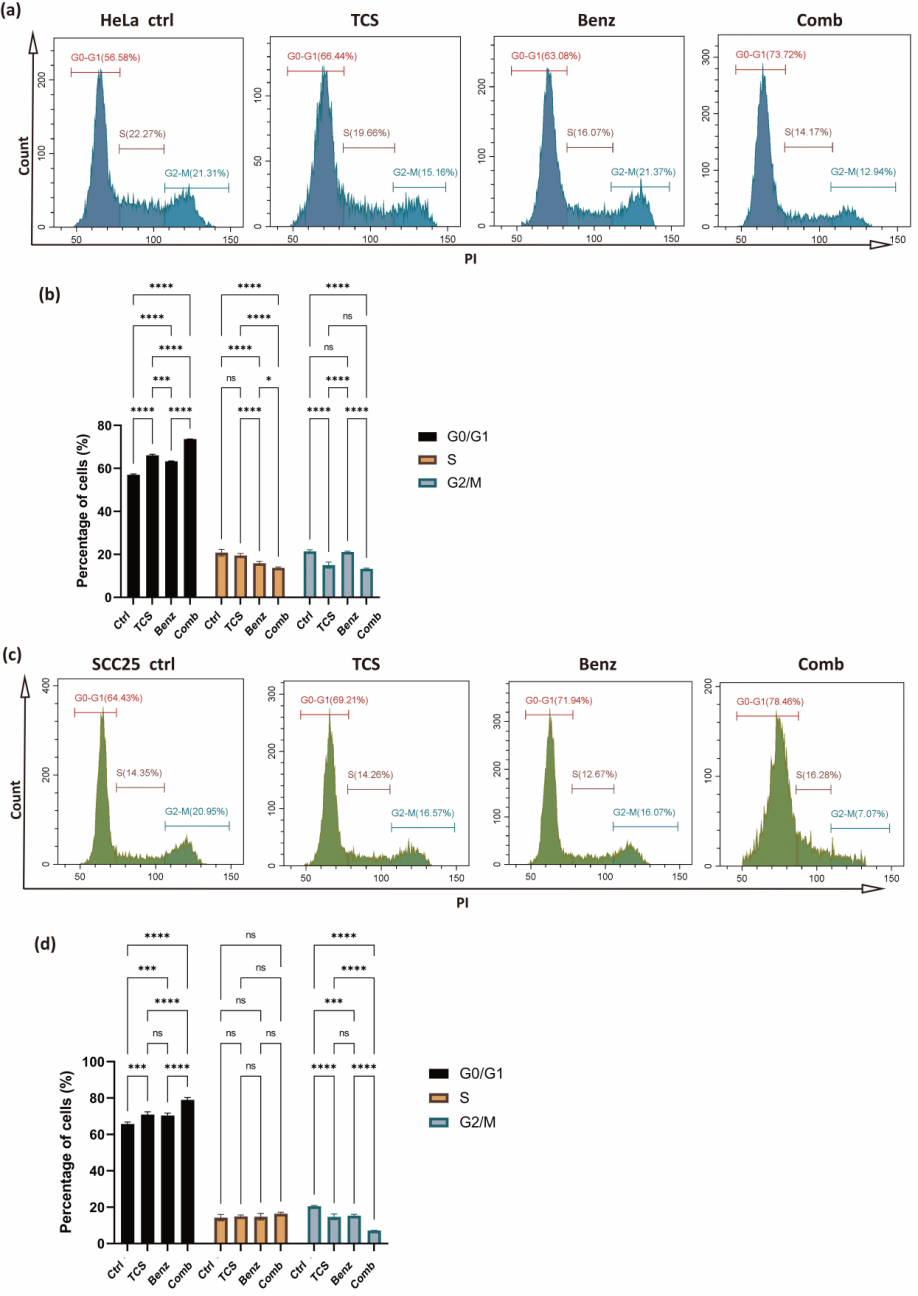
Cell cycle analysis after 24 h of treatment. HeLa cells were treated with 25 μg/mL TCS and 25 μM Benz, either alone or in combination. SCC25 cells were treated with 12.5 μg/mL TCS and 12.5 μM Benz, either alone or in combination. **(a)** Flow cytometry results showing cell cycle distribution in HeLa cells. **(b)** The cell percentage of G0/G1, S and G2-M phases were quantified and plotted as histogram in HeLa cells. **(c)** Flow cytometry results depicting cell cycle distribution in SCC25 cells. **(d)** Quantification of cell cycle distribution in SCC25 cells. Results were presented as mean ± SE (*n* = 3); ns indicates non-significant, * represents *P*< 0.05, ***represents *P*<0.001, **** represents *P*< 0.0001.

To investigate the impact of the combined use of TCS and Benz on growth and mitosis in HeLa and SCC25 cells, the cell cycle distribution was analysed using a Cyto-Flex flow cytometer. Compared to the control group and single treatment groups, the combination treatment group exhibited significant increase in the G0/G1 phase, accompanied by a decrease in the S and G2/M phases, suggesting induction of cell cycle arrest at the G0/G1 phase while inhibition of the transition to the G2/M phase in HeLa cells (Figures 5a and 5b). Similarly, in SCC25 cells, the combination treatment also led to cell cycle arrest at the G0/G1 phase and blocking of G2/M phase, with the exception that the effects on the S phase were not statistically significant (Figures 5c and 5d). These results revealed that the combination of TCS and Benz exerted a regulatory effect on the cell cycle progression, potentially contributing to the inhibition of cell proliferation in both HeLa and SCC25 cells.

### TCS combined with Benz to elevate cellular ROS accumulation and trigger mitochondria associated cell apoptosis

Previous research has shown that inhibiting aerobic glycolysis in cancer cells may cause damage to mitochondria through ROS, ultimately leading to increased apoptosis [34-36]. TCS has also been found to induce ROS as an anticancer mechanism [37-39].

**Figure 6.**
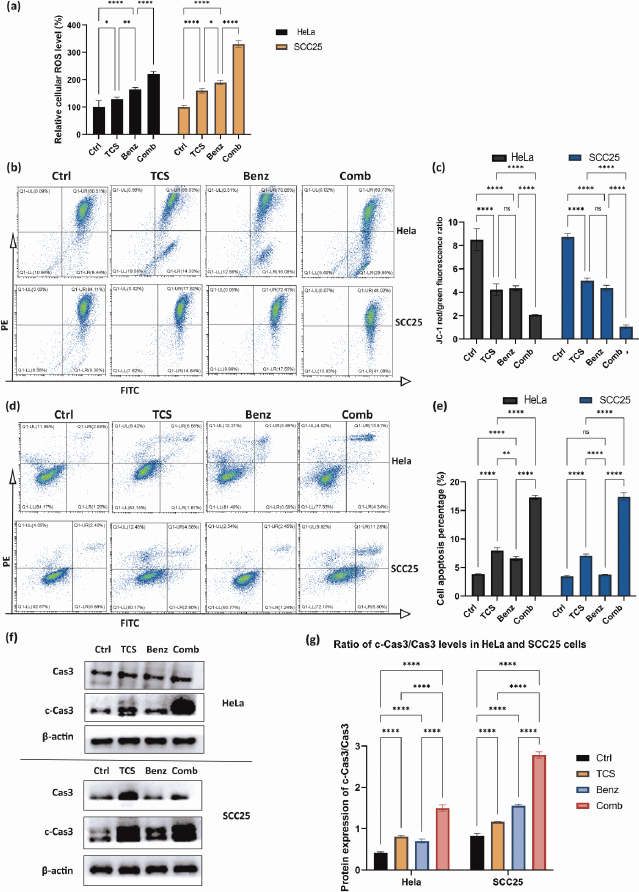
Cellular ROS, mitochondrial membrane potential (MMP), and apoptosis in HeLa and SCC25 cell models. HeLa cells were treated with 25 μg/mL TCS and 25 μM Benz, alone or in combined. SCC25 cells were treated with 12.5 μg/mL TCS and 12.5 μM Benz, alone or in combined. **(a)** Cellular ROS amounts were measured in both cell lines after 4 h drug treatment. **(b)** Cells were exposed to 24 h treatment, followed by flow cytometry assay to detect the MMP changes which were indicated by the ratio of JC-1 aggregate (PE-red)/JC-1 monomer (FITC-green) fluorescence signal. **(c)** Quantification results of MMP. **(d)** Flow cytometry to determine the percentage of apoptotic cells after 24 h treatment. Late apoptotic cells are shown in the upper right quadrant, and early apoptotic cells are shown in the lower right quadrant. **(e)** Quantification results of cell apoptosis percentage. **(f, g)** Western blotting to detect apoptotic protein Caspase 3 (Cas3) and its cleavage in HeLa and SCC25 cells. Data were presented as mean ± SE (*n* = 3); ns indicates non-significant, * represents *P*< 0.05, ** represents *P*<0.01, **** represents *P*< 0.0001.

As shown in Figure 6a, combined use of TCS and Benz resulted in dramatic increase in intracellular ROS, suggesting potential metabolic stress in both HeLa and SCC25 cells. JC1 staining assays further showed mitochondrial membrane potential (MMP) depolarization under combination treatments in both cell lines (Figure 6b and 6c). Next, flow cytometry after Annexin-V/PI dual staining revealed that the combination of TCS and Benz induced markedly early and late apoptosis in both cells, as compared with single compound treatment (Figures 6d and 6e). Furthermore, significant increase of pro-apoptotic protein, cleaved Caspase 3 (c-Cas3), and distinctively elevated ratio of c-Cas3/Cas3 were detected *via* western blotting under this combination treatment in both HeLa and SCC25 cells, indicating apoptosis cascades stimulation and enhanced cell apoptotic activities (Figure 6f and 6g).

### TCS treatment alone induced activation of NF-κB/ERK survival signalling

**Figure 7.**
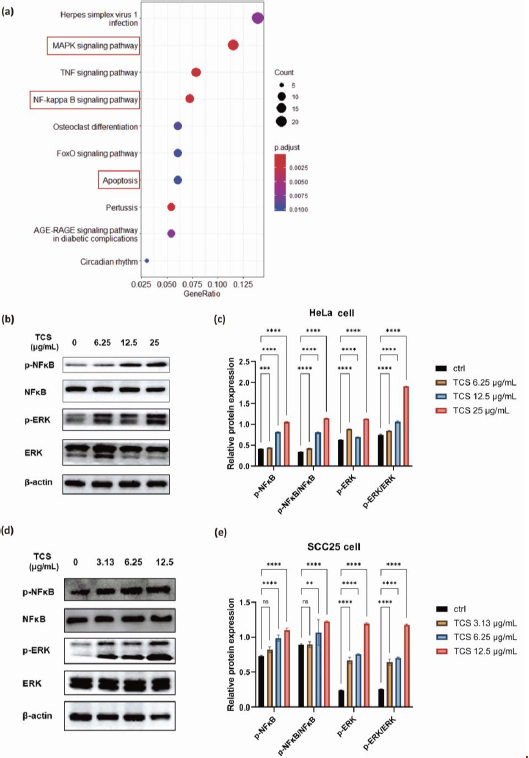
Effect of TCS treatment alone on NF-κB/ERK related gene/protein expressions. **(a)** KEGG enrichment analysis of RNA-sequencing data in HeLa cells with 80ng TCS treatment for 24h. **(b)** Western blot results of NF-κB, p-NF-κB, ERK, p-ERK protein expressions. HeLa cells were exposed to a serial of TCS treatments at 6.25, 12.5 and 25 μg/mL, respectively, for 24 h. **(c)** Quantification results of relative protein expression level in HeLa cells. **(d)** Western blot results of NF-κB, p-NF-κB, ERK, p-ERK protein expressions. SCC25 cells were exposed to a serial of TCS treatments at 3.13, 6.25 and 12.5 μg/mL, respectively, for 24 h. **(e)** Quantified results of relative protein expressions in SCC25 cells. Data were presented as mean ± SE (*n* = 3); ns indicates non-significant, ** represents *P*<0.01, ***represents *P*<0.001, **** represents *P*< 0.0001.

To investigate the transcriptome changes and explore the intricate molecular mechanisms under TCS single treatment, RNA-sequencing analysis was performed on HeLa cells exposed to 80 ng TCS for 24 h. The Kyoto Encyclopedia of Genes and Genomes (KEGG) enrichment analysis showed TCS treatment alone induced apoptosis and activation of cancer survival signalling including MAPK and NF-κB pathways (Figure 7a).

Subsequent western blotting results, which showed significantly promoted phosphorylation of NF-κB p65 and p44/42 MAPK (Erk1/2), indicating the activations of MAPK/ERK and NF-κB signalling pathways were induced by TCS treatment alone in HeLa cells (Figures 7b and 7c). Intriguingly, similar phenomena were also observed in SCC25 cells (Figures 7d and 7e).

### TCS combined with Benz inhibited NFκB/ERK axis in HeLa and SCC25 cells

**Figure 8.**
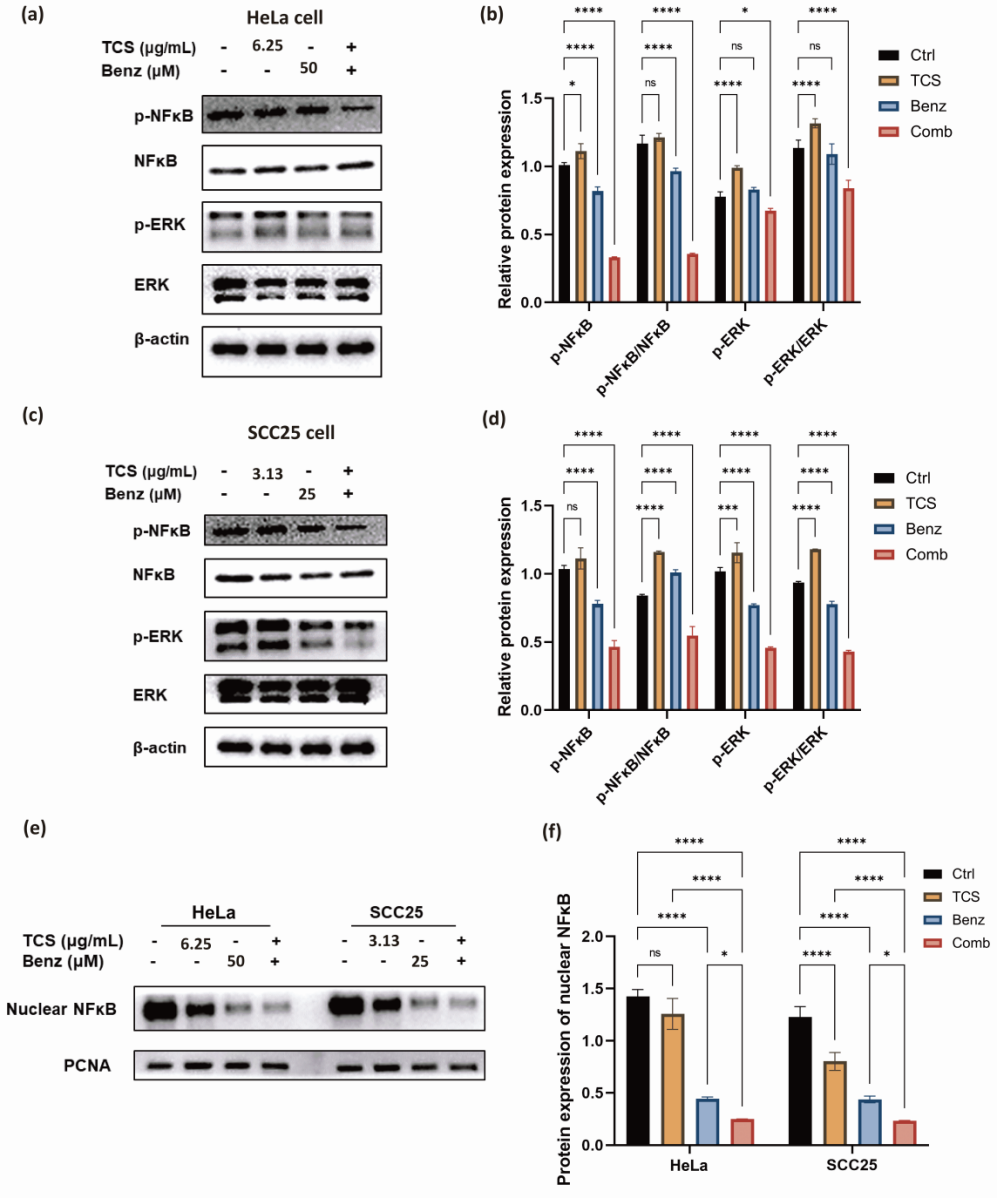
Effect of TCS combined with Benz treatment on NF-κB/ERK related protein expression. HeLa cells were treated with 6.25 μg/mL TCS and 50 μM Benz, alone or in combined, for 24 h. SCC25 cells were treated with 3.13 μg/mL TCS and 25 μM Benz, alone or in combined, for 24 h. **(a)** Western blot results of NF-κB, p-NF-κB, ERK, p-ERK protein expressions in HeLa cells. **(b)** Quantification results of relative protein expression in HeLa cells. **(c)** Western blot results of NF-κB, p-NF-κB, ERK, p-ERK protein expressions in SCC25 cells. **(d)** Quantified results of relative protein expression in SCC25 cells. **(e)** Examination of nuclear NF-κB protein expression within nuclear extracts of HeLa and SCC25 cells. **(f)** Quantified results of nuclear NF-κB protein level in HeLa and SCC25 cells. Data were presented as mean ± SE (*n* = 3); ns indicates non-significant, * represents *P*< 0.05, ***represents *P*<0.001, **** represents *P*< 0.0001.

Next, the protein expressions of phospho-NF-κB, phospho-ERK, as well as the ratio of p-NF-κB / NF-κB and p-ERK/ERK were found to be significantly down-regulated under combined use of TCS and Benz in HeLa cells (Figures 8a and 8b).

Consistently, the suppression the NF-κB/ERK axis were also shown in SCC25 cells under combination strategy (Figures 8c and 8d). Additionally, nuclear NF-κB protein levels were prominently decreased under combination treatment in both cell lines as revealed by western blotting of the isolated nuclear fractions, suggesting inhibition of nuclear translocation of NF-κB (Figures 8e and 8f). Taken together, these results indicated that TCS combined with Benz had great potential to interfere two pivotal pro-survival signalling activations, namely NF-κB and MAPK/ERK in both HeLa and SCC25 cells, mainly by inhibiting NF-κB and ERK phosphorylation, as well as restraining the nuclear translocation of NF-κB.

### Co-administration of TCS and Benz effectively inhibited tumour growth in SCC25 cell xenograft mouse model

**Figure 9.**
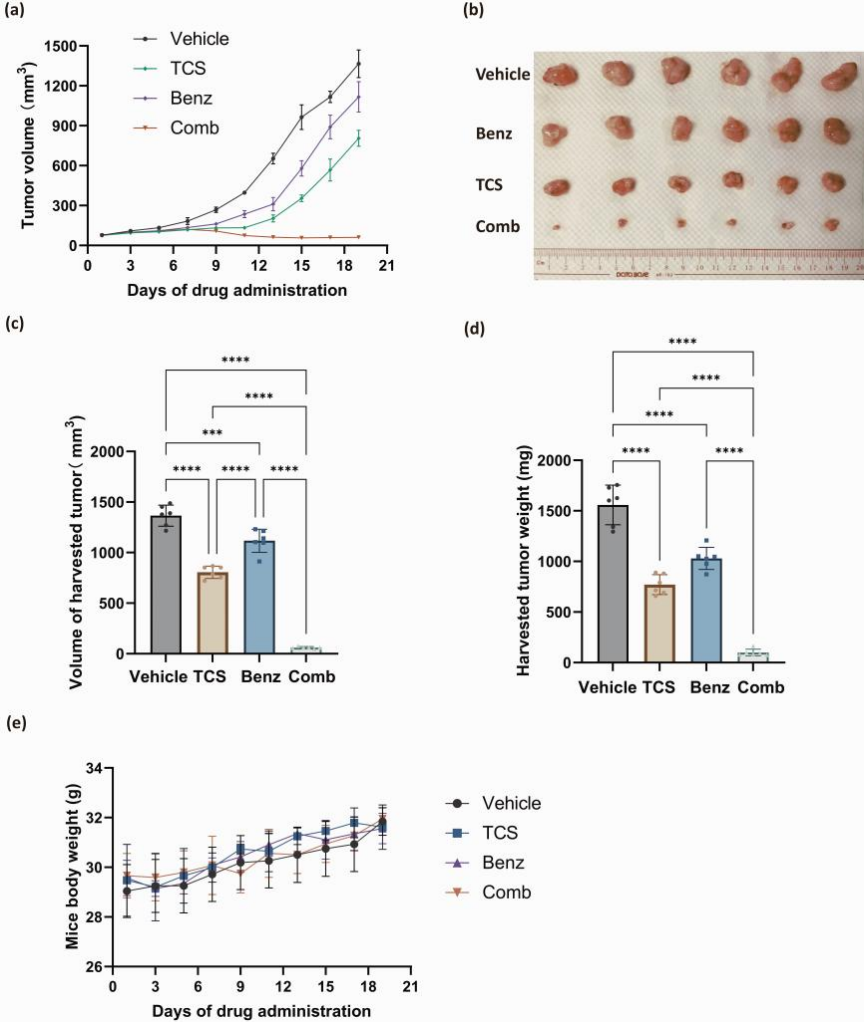
The *in vivo* evaluation of anti tumour efficacy of TCS and Benz combination in SCC25 cell xenograft mouse model. Nude mice aged between 6 and 8 weeks were randomly divided into four groups, with each group consisting of six mice: the vehicle group (administered with PBS containing 10 % PEG), the TCS group (1 mg/kg), the Benz group (500 mg/kg), and the combination group (1mg/kg TCS + 500 mg/kg Benz). **(a)** The tumour growth curve was recorded for each administration group over a period of 19 days. **(b)** At the end of the drug administration period, photographs of the harvested tumour s were taken. **(c, d)** The volume and weight of the harvested tumour s were measured. **(e)** Changes in the body weight of the animals were monitored throughout the 19-day drug administration period. Data were presented as mean ± SD (*n* = 6); ***represents *P*<0.001, **** represents *P*< 0.0001.

Overall, our *in vitro* data showed that Benz significantly enhanced anticancer effects of TCS in both HeLa and SCC25 cells. Yet, SCC25 cells were more sensitive to this combination. To this end, the combination of TCS and Benz were evaluated *in vivo* using a SCC25 cell xenograft mouse model. The administrations of TCS and Benz, either alone or in combination, were carried out every other day for a period of 19 days. The dosage used was 1 mg/kg for TCS and 500 mg/kg for Benz. As shown in Figure 9a, the combined use of TCS and Benz significantly suppressed the tumour growth, outperforming the single use of either TCS or Benz.

The analysis of tumour volume and weights on the final day of drug dosing also indicated the potent anti tumour efficacy of this drug combination (Figures 9b, 9c and 9 d). Moreover, the daily activities and weight changes of the animals were minimally affected by the combined application, suggesting negligible treatment related systemic toxicity (Figure 9e). These results further validated the plausible tumour suppressing efficacy of TCS and Benz combination strategy.

## Discussion

As far as we are aware, the use of TCS in combination with HK2 inhibition, or glycolytic inhibition in general, had not been undertaken. Our findings suggested that this combination might offer a unique and innovative therapeutic strategy for enhancing the anticancer effects of TCS. By selectively targeting the aberrant glycolysis in cancer cells, this approach offers great promise for improving cancer treatment outcomes.

Prior studies have highlighted the critical role of HK2 in promoting tumour growth and progression by facilitating increased glycolytic activity [10,32,33]. Consistent with previous studies, our results confirmed the significant upregulation of HK2 expression in malignant tumours, emphasizing its potential as a therapeutic target. Importantly, we found that single use of TCS significantly enhanced HK activities, which offered an exciting opportunity for combined use of TCS and HK2 inhibitor to enhance anticancer effects. In the present study, the combination of TCS with Benz exhibited strong anticancer synergism in both HeLa and SCC25 cell models, suggesting HK2 inhibition can effectively potentiate the anticancer effects of TCS.

Cancer cells benefit from the Warburg effect *via* increasing glucose uptake and stimulating lactate production [7,40,41]. The addition of Benz in TCS treatment significantly decreased cellular glycolysis and ATP levels. This reduction indicates the disruption of vital metabolic pathways in cancer cells, leading to cellular stress and compromised survival. Moreover, the combination therapy exhibited a dual impact on cancer cell behaviour by lowering lactate secretion and suppressing colony-forming ability. In particular, we found that the combination of TCS and Benz induced cell cycle arrested at the G0/G1 phase and blocked the transition into the G2/M phase in both HeLa and SCC25 cells, which further substantiated its inhibitory effects on cancer cell growth and proliferation [42].

In addition to its effects on cellular metabolism and cell cycle regulation, the combination of TCS and Benz exhibited profound impacts on cellular apoptosis. The treatment increased cellular ROS accumulation and triggered mitochondria-associated cell apoptosis. These observations suggest that the combination therapy exerts its potent anticancer effects by inducing oxidative stress and promoting programmed cell death.

TCS treatment alone activated the NF-κB/ERK signalling pathway, which can be rationalized as a survival mechanism of the cancer cells in order to overcome the external assault induced by TCS [43,44]. Previous study suggested that TCS-induced autophagic death of cancer cells might be related to the activation of NF-κB pathway [45]. Despite the interplays between autophagy, cell death and NF-κB pathway are not fully understood, the activation of NF-κB pathway appears to play an irreplaceable role in promoting cancer cell proliferation [46]. We found that combined use of TCS and Benz effectively inhibited the NF-κB/ERK axis, thereby counteracting the pro-survival signalling pathways activated by TCS alone. The decrease in nuclear NF-κB protein levels, as observed through western blotting analysis, indicated that the combination treatment inhibited the nuclear translocation of NF-κB, effectively contributing to the anticancer effects.

Based on prior *in vivo* study, Benz was shown to be a safe agent as no significant weight loss or changes in tissue morphology were observed in mice with daily *i.p.* injections of up to 600 mg/kg body weight of Benz for 16 days [25]. Recent studies suggested that high doses of TCS administration alone can be toxic [47,48]. Minimal side effects were shown by *i.p.* administration of low dose of 0.5-1.0 mg/kg TCS for 19 days, but there was a tendency for mice to lose weight at TCS doses up to 2.0 mg/kg [18, 23]. Importantly, our *in vivo* experiments using a SCC25 cell xenograft mouse model clearly demonstrated that the combined use of TCS and Benz effectively inhibited tumour growth at a low dose (1.0 mg/kg TCS - 500 mg/kg Benz), leading to tumour regression without any observable side effects. These results clearly supported the translational potential of the TCS-Benz combo in anticancer treatment.

## Conclusions

Our findings highlighted the strong synergistic anticancer effects by combining TCS with Benz in HeLa and SCC25 cancer cell models. The combination of TCS and Benz demonstrated great potential for interfering with pro-survival signalling pathways, namely, NF-κB and MAPK/ERK, to facilitate cancer cell killing. Moreover, the observed effects on glycolysis, ROS accumulation, cell cycle regulation, and apoptosis induction collectively contributed to the multifaceted mechanisms through which the TCS-Benz combo exerted its potent anticancer effects. This study provides valuable insights for potential therapeutic strategies that can enhance the anticancer efficacy of TCS by targeting HK2, suggesting that modulating aberrant glycolysis holds a promise to enhance the sensitivity of cancer cell to TCS-based therapeutic strategy.
